# The association of leisure‐time physical activity and walking during commuting to work with depressive symptoms among Japanese workers: A cross‐sectional study

**DOI:** 10.1002/1348-9585.12120

**Published:** 2020-05-05

**Authors:** Kota Fukai, Keisuke Kuwahara, Sanmei Chen, Masafumi Eguchi, Takeshi Kochi, Isamu Kabe, Tetsuya Mizoue

**Affiliations:** ^1^ Department of Preventive Medicine Tokai University School of Medicine Isehara City Japan; ^2^ Teikyo University Graduate School of Public Health Itabashi‐ku Japan; ^3^ Department of Epidemiology and Prevention Center for Clinical Sciences National Center for Global Health and Medicine Shinjuku‐ku Japan; ^4^ Department of Health Administration Furukawa Electric Corporation Japan

**Keywords:** cross‐sectional study, depressive symptoms, leisure‐time physical activity, sedentary behaviors, walking during commuting to work

## Abstract

**Objective:**

To examine whether the cross‐sectional association of leisure‐time physical activity and walking during commuting to work with depressive symptoms depends on the level of work‐related physical activity among Japanese workers.

**Methods:**

Participants were 2024 workers aged 19‐69 years in two manufacturing companies in Japan. Leisure‐time physical activity and walking during commuting to work were ascertained via a self‐administered questionnaire. Depressive symptoms were assessed using the Center for Epidemiologic Studies Depression (CES‐D) scale. The odds ratio (OR) of depressive symptoms (CES‐D score ≥16) was estimated by using multiple logistic regression with adjustment for covariates.

**Results:**

Leisure‐time physical activity was inversely associated with depressive symptoms; multivariable‐adjusted ORs (95% confidence intervals) of having depressive symptoms for leisure‐time physical activity were 1.00 (reference), 0.85 (0.64, 1.12), 0.69 (0.51, 0.94), and 0.59 (0.44, 0.80) for 0, >0 to <3.0, 3.0 to <10.0, and ≥10.0 MET‐h/wk, respectively (*P* for trend <.001). This inverse trend for leisure‐time physical activity was clearer among individuals who had low physical activity at workplace (less than 7.0 MET‐h/d). For walking to work, such an inverse association was not observed.

**Conclusion:**

Leisure‐time physical activity was associated with fewer depressive symptoms, especially in workers with low work‐related physical activity.

## 1 INTRODUCTION

Depressive disorders are a significant public health problem among working‐age adults.^1^ In Japan, the 12‐month prevalence of major depressive disorders among 20 year olds or older was estimated to be 2.2% based on the World Mental Health Japan Survey conducted in 2002‐2006.^2^ In a survey involving 24 896 individuals randomly selected from among 300 communities throughout Japan, 25.6% of males and 29.5% of females aged 20‐59 years had depressive symptoms.^3^ Depression is also the leading cause of long‐term sickness absence and presenteeism among working adults in many developed countries.^4^ In Japan, the workplace costs of long‐term sickness absence and presenteeism due to depression was estimated up to US$6912 million per year in 2008 based on the national health statistics.^5^ Organizations are now widely required to weigh in evidence‐based workplace interventions for their employees’ health including mental health.^6^

The promotion of physical activity during leisure‐time is recommended owing to the beneficial effect not only on physical health but also on mental health as well.^7^ A meta‐analysis has shown that higher physical activity levels are associated with a decreased risk of developing depression.^8^ A recent multi‐cohort study in Europe showed that higher physical activity levels are associated with a lower risk of sickness absence due to depressive disorders.^9^ In Japan, a cohort study among Japanese workers showed a risk reduction of depressive symptoms associated with high leisure‐time exercise, especially at high intensity.^10^

Nevertheless, it would be quite challenging for workers to increase physical activity levels during leisure‐time due to long working hours^11^ or tiredness.^12^ Walking is a beneficial exercise that can be readily incorporated into daily commuting for workers^13^ and has minimal risk of adverse effects.^14^ Thus, it is interesting to see whether walking during commuting to work can help prevent depression. However, data on the associations of commuting activity with depressive symptoms are scarce for working populations.^15^, ^16^ In addition, the association of commuting physical activity with depressive symptoms may differ between those who engaged in high work‐related physical activity and those who are not. However, to our knowledge, no studies have investigated the potential moderating role of work‐related physical activity in the association between commuting physical activity and depressive symptoms.

The purpose of the present study was to clarify the association between physical activities of both leisure‐time physical activity and walking during commuting to work on depressive symptoms among Japanese workers. We additionally performed subgroup analysis stratified by work‐related physical activity.

## 2 METHODS

### 2.1 Study design and participants

This study used the baseline data from the Furukawa Nutrition and Health Study, a part of the Japan Epidemiology Collaboration on Occupational Health Study. The details of the study procedure have been explained elsewhere.^11^, ^12^, ^17^ Briefly, the baseline survey was conducted during periodic health examination in April 2012 (factory A in Chiba Prefecture) and May 2013 (factory B in Kanagawa Prefecture) among employees of a manufacturing company and its affiliates in Japan. All employees (n = 2828) were invited to participate in the survey and asked to fill out two types of survey questionnaires (one for diet and the other for health‐related lifestyles including physical activity and depressive symptoms). We also obtained health examination data regarding anthropometric and biochemical data and disease history. The study protocol was approved by the ethics committee of the National Center for Global Health and Medicine, Japan. Written informed consent was obtained from each participant prior to the survey.

Of the 2828 health examination attendees (women, 11%), 2162 (1930 men and 232 women aged 18‐70 years) agreed to participate in the study with a response rate of 76%. Of the 2162 workers, we excluded 100 participants with a history of the following diseases: cancer (n = 20), cardiovascular diseases (n = 25), chronic hepatitis (n = 2), kidney disease including nephritis (n = 11), pancreatitis (n = 3), and mental disorders such as depression and neurotic disorder (n = 45). Some participants had two or more of these diseases. We excluded these participants to avoid reverse causality because such conditions might affect physical activity habits or depressive status. Of the remaining 2062, we excluded 38 participants who had missing data of covariates used for the present analysis, leaving 2024 participants (1809 men and 215 women) aged 19‐69 years for analysis.

### 2.2 Assessment of physical activity

Physical activity was assessed according to the domain using the self‐administered questionnaire. For leisure‐time physical activity, participants reported frequency (none, 1‐3 times per month, 1‐2 times per week, 3 or 4 times per week, 5 or 6 times per week, or almost every day) and duration (none, >0 to <30 minutes, 30 minutes to <1 hour, 1 to <2 hours, 2 to <3 hours, 3 to <4 hours, or ≥4 hours) of the activity in the past 12 months by the intensity: light intensity (walking for pleasure, hiking for pleasure, callisthenics, golf, lawn and garden for pleasure, etc), moderate intensity (jogging, swimming, skiing, dancing, moderate ball games, etc), and vigorous intensity (fighting sports, ball games, running, etc). For work‐related physical activity, participants were asked about the average time spent in the activity per day (none, >0 to <30 minutes, 30 minutes to <1 hour, 1 to <3 hours, 3 to <5 hours, 5 to <7 hours, 7 to <9 hours, or ≥9 hours) according to the five intensities: sitting, standing, walking, cycling, heavy physical work.

We assigned the value of metabolic equivalents (METs) for each activity as follows: for leisure‐time activity, two METs for light intensity, five METs for moderate intensity, and eight METs for vigorous intensity, and for work‐related physical activity, two METs for standing, three METs for walking and cycling, and six METs for heavy physical work. The total volume of leisure‐time physical activity (MET‐h/wk) and work‐related physical activity (MET‐h/d) were calculated by multiplying the intensity of each type of activity, time spent in the activity, and frequency for leisure‐time physical activity. For calculation, the midpoint of the time range and frequency for each category were assigned. Sedentary time was not included in the calculation. After calculation, participants were categorized into four groups for leisure‐time activity, approximately according to the quartiles of the volume of leisure‐time physical activity: none (0 MET‐h/wk), low (>0 to <3.0 MET‐h/wk), medium (3.0 to <10.0 MET‐h/wk), and high (≥ 10.0 MET‐h/wk).

For commuting activity, participants reported about the time spent in walking to work (one‐way) using the following response options: none, >0 to <10 minutes, 10 to <15 minutes, 15 to <30 minutes, 30 to <45 minutes, or ≥45 minutes. Participants were re‐categorized into four groups for walking during commuting to work according to the distribution: none, >0 to <15 minutes, 15 to <30 minutes, and ≥30 minutes. These cutoffs were determined based on previous Japanese studies.^18^, ^19^

### 2.3 Assessment of depressive symptoms

Depressive symptoms were assessed using the Center for Epidemiologic Studies Depression (CES‐D) scale.^20^ The CES‐D scale consists of 20 items addressing six typical depression symptoms, including depressed mood, guilt or worthlessness, helplessness or hopelessness, psychomotor retardation, loss of appetite, and sleep disturbance experienced during the preceding week. Each item is scored on a scale of 0–3 (“0 no,” “1 sometimes,” “2 often,” and “3 always”) according to the frequency of the symptom and calculated total CES‐D score for each participant, ranging from 0 to 60. The criterion validity of the CES‐D scale has been well established in both Western^20^ and Japanese^21^ participants. Also, the internal consistency of the depression score was high; Cronbach's alpha was 0.865 for the present data. CES‐D score of 16 or higher was considered as having depressive symptoms.^20^

### 2.4 Other variables

Body height and weight were measured in a standardized procedure.^22^ Body mass index (BMI) was calculated as weight in kilograms divided by the square of height in meters. Alcohol drinking, smoking, night and rotating shift work, overtime work, job grade, sleep duration, marital status were assessed via the survey questionnaire.^22^, ^23^ Total amount of alcohol consumption was calculated using data on weekly frequency and daily amount of consumption of alcoholic beverages. Psychological work environment was assessed via the Job Content Questionnaire,^24^ and job strain was defined as the ratio of demands to job control.^25^

### 2.5 Statistical analysis

The characteristics of the participants were expressed as the means (SD) and percentages for continuous variables and categorical variables, respectively. To examine the association between leisure‐time physical activity levels and depressive symptoms, we performed multiple logistic regression analysis to calculate the odds ratios (ORs) and 95% confidence intervals (CIs) of having depressive symptoms according to leisure‐time physical activity levels, using the “none” category as reference. Similarly, we conducted the same analysis for walking during commuting to work, using the “none” category as reference. In model 1, we adjusted for age (in years, continuous), sex (male or female), worksite (factory A or B). In model 2, we further adjusted for BMI (kg/m^2^, continuous), alcohol drinking (non‐drinker, drinker consuming <1, 1 to <2, or ≥2 *go* of Japanese sake equivalent per day), and smoking (never smoker, quitter, current smoker consuming <20 cigarettes/d, or current smoker consuming ≥20 cigarettes/d), night or rotating shift work (yes or no), overtime work (<10, 10 to <30, or ≥30 hours per month), job grade (low, middle, or high), sleep duration on weekdays (<5, 5 to <6, 6 to <7, or ≥7 h/d), marital status (married or other), job strain (quartile). In model 3, daily work‐related physical activity (MET‐h/d, continuous) and other types of physical activity were mutually adjusted. That is, walking during commuting to work was adjusted for leisure‐time physical activity and leisure‐time physical activity was adjusted for walking during commuting to work. Linear trend for the association of leisure‐time physical activity or walking during commuting to work was tested by treating these variables as continuous ones.

For subgroup analysis, we performed the same analysis stratified by the median of work‐related physical activity (7 MET‐h/d). In model 3, other types of physical activity were mutually adjusted for each model. That is, walking during commuting to work was adjusted for the model of leisure‐time physical activity and leisure‐time physical activity was adjusted for the model of walking during commuting to work. Test for interaction was conducted by using the likelihood ratio test. An interaction term was generated by multiplying the dichotomized variable of daily work‐related physical activity by four categories of leisure‐time physical activity or walking during commuting to work (treated as continuous variables, with ordinal numbers assigned to each level) and added to model 3. Likewise, we examined the interaction of sex and age, both with leisure‐time physical activity and walking during commuting to work.

A two‐sided *P* value <.05 was considered statistically significant in all analyses. All analyses were performed using Statistical Analysis System (SAS) Software Version 9.4 (SAS Institute).

## 3 RESULTS

In total, 559 participants (27.6%) were identified as having depressive symptoms (27.6% for male and 27.4% for female). Participant characteristics by leisure‐time physical activity levels are presented in Table 1. Participants who engaged in more physical activity during leisure‐time tended to be male, had a high job position, were married, were less likely to smoke, and were engaged in shift work. BMI and job strain did not differ by the dose of leisure‐time physical activity. Characteristics by walking during commuting to work are available in Table S1.

**TABLE 1:** Characteristics of participants by leisure‐time physical activity (N = 2024)

	Leisure‐time physical activity (MET‐h/w)			
	None	0 to <3.0	≥3.0 to<10.0	≥10
Number of participants	532 (26.3)	527 (26.0)	436 (21.5)	529 (26.1)
Male sex	446 (83.8)	464 (88.0)	404 (92.7)	495 (93.6)
Age, years	41.3 ± 9.2	42.7 ± 10.0	42.7 ± 10.7	42.3 ± 10.5
BMI, kg/m^2^	23.1 ± 3.7	23.3 ± 3.5	23.3 ± 3.3	23.2 ± 2.9
CES‐D score, points	14.0 ± 7.9	12.3 ± 7.1	11.9 ± 7.6	10.7 ± 7.3
Job strain, score	0.51 ± 0.14	0.49 ± 0.11	0.47 ± 0.11	0.47 ± 0.11
Current smoker	205 (38.5)	130 (24.7)	135 (31.0)	115 (21.7)
Heavy alcohol drinker^a^	50 (9.4)	34 (6.5)	41 (9.4)	44 (8.3)
High job position	130 (24.4)	171 (32.5)	143 (32.8)	182 (34.4)
Shift work	156 (29.3)	91 (17.3)	76 (17.4)	86 (16.3)
Long overtime work (>45 h/mo)	28 (5.3)	27 (5.1)	22 (5.0)	19 (3.6)
Short sleep (<5 h/d)	56 (10.5)	30 (5.7)	29 (6.7)	41 (7.8)
Married	304 (57.1)	374 (71.0)	300 (68.8)	358 (67.7)
High work‐related physical activity^b^	295 (55.5)	230 (43.6)	209 (47.9)	272 (51.4)
Walking during commuting to work (per one way)				
None	371 (69.7)	345 (65.5)	289 (66.3)	314 (59.4)
>0 to <15 min	108 (20.3)	123 (23.3)	94 (21.6)	117 (22.1)
15 to <30 min	40 (7.5)	41 (7.8)	31 (7.1)	45 (8.5)
≥30 min	13 (2.4)	18 (3.4)	22 (5.0)	53 (10.0)

Note

Data are shown as mean ± standard deviation for continuous variables and number (percentages) for categorical variables.

Abbreviations: BMI, body mass index; CES‐D, Center for Epidemiologic Studies Depression; MET, metabolic equivalent.

^a^ ≥ 2 go of Japanese sake equivalent, 1 go of Japanese sake contains approximately 23 g of ethanol.

^b^ ≥ 7 MET‐h/d or more of work‐related physical activity.

As shown in Table 2, leisure‐time physical activity was inversely associated with depressive symptoms in the total population. The multivariable‐adjusted ORs (95% CIs) of depressive symptoms were 1.00 (reference), 0.85 (0.64, 1.12), 0.69 (0.51, 0.94), and 0.59 (0.44, 0.80) for 0, >0 to <3.0, 3.0 to <10.0, and ≥10.0 MET‐h/wk of leisure‐time physical activity, respectively (*P* for trend <.001) after adjusting for potential confounders, including commuting physical activity (model 3). *P* for interaction were .64 and .30 for sex and age, respectively, of leisure‐time physical activity. When participants were divided by work‐related physical activity level, the association among participants with high work‐related physical activity was less clear than that among those with low work‐related physical activity, although the interaction by work‐related physical activity on the association between leisure‐time physical activity and depressive symptoms did not reach statistical significance level (*P* for interaction = .16). Among participants with low level of work‐related physical activity, the multivariable‐adjusted ORs were 1.00 (reference), 0.58 (0.39, 0.88), 0.41 (0.25, 0.65), and 0.43 (0.28, 0.69) for 0, >0 to <3.0, 3.0 to <10.0, and ≥10.0 MET‐h/wk of leisure‐time physical activity, respectively (*P* for trend <.001), whereas the corresponding values were 1.00 (reference), 1.16 (0.78, 1.72), 1.07 (0.71, 1.62), and 0.69 (0.46, 1.05) among participants with high work‐related physical activity (*P* for trend = .10).

**TABLE 2:** Odds ratios of having depressive symptoms by leisure‐time physical activity (N = 2024)

	ORs (95% CI) by leisure‐time physical activity (MET‐h/w)				
	None	0 to <3.0	≥3.0 to <10.0	≥10.0	*P* for trend^d^
Total population					
No. of subjects	532	527	436	529	
No. of prevalent cases (%)	186 (35.0)	150 (28.5)	107 (24.5)	116 (21.9)	
Unadjusted model	1.00 (Reference)	0.74 (0.57, 0.96)	0.61 (0.46, 0.80)	0.52 (0.40, 0.69)	<.001
Model 1^a^	1.00 (Reference)	0.76 (0.58, 0.98)	0.60 (0.45, 0.80)	0.51 (0.39, 0.68)	<.001
Model 2^b^	1.00 (Reference)	0.85 (0.65, 1.13)	0.70 (0.51, 0.95)	0.59 (0.44, 0.80)	<.001
Model 3^c^	1.00 (Reference)	0.85 (0.64, 1.12)	0.69 (0.51, 0.94)	0.59 (0.44, 0.80)	<.001
Work‐related physical activity					
Low group					
No. of subjects	237	297	227	257	
No. of prevalent cases (%)	88 (37.1)	74 (24.9)	45 (19.8)	52 (20.2)	
Model 3^c^	1.00 (Reference)	0.58 (0.39, 0.88)	0.41 (0.25, 0.65)	0.43 (0.28, 0.69)	<.001
High group					
No. of subjects	295	230	209	272	
No. of prevalent cases (%)	98 (33.2)	76 (33.0)	62 (29.7)	64 (23.5)	
Model 3^c^	1.00 (Reference)	1.16 (0.78, 1.72)	1.07 (0.71, 1.62)	0.69 (0.46, 1.05)	.10
	*P* for interaction^e^ = .16				

Abbreviations: BMI, body mass index; CI, confidence intervals; MET, Metabolic equivalent; OR, odds ratio.

^a^ Adjusted for age (in years, continuous), sex (male or female), site (factory A or B).

^b^ Adjusted for factors in Model 1 plus BMI (kg/m^2^, continuous), smoking (never smoker, quitter, current smoker consuming 1 to <20, or ≥20 cigarettes per day),alcohol consumption (non‐drinker, drinker consuming <1, 1 to <2, or ≥2 *go* of Japanese sake equivalent per day, where 1 *go* of Japanese sake contains approximately 23 g of ethanol), sleep duration on weekdays (<5 h per night, 5 to <6 h per night, 6 to <7 h per night, or ≥7 h per night), marital status (married or other), night or rotating shift work (yes or no), overtime work (<10 h per month, 10 to <30 h per month, or ≥30 h per month), job grade (low, middle, or high), job strain (quartile).

^c^ Adjusted for factors in Model 2 plus daily work‐related physical activity (MET‐h/d, continuous) and walking during commuting to work (none, >0 to <15 min, 15 to <30min, ≥30 min per one‐way).

^d^ The trend test were calculated for the associations between categories of leisure‐time physical activity converted into continuous variables and depressive symptoms.

^e^ Interaction between work‐related physical activity and leisure‐time physical activity was calculated by adding interaction terms in the model.

As shown in Table 3, although the ORs of depressive symptoms tended to decrease with increasing categories of walking during commuting to work, it was not statistically significant in the total population. Even after the participants were stratified by the levels of work‐related physical activity, the associations were not significant (*P* for interaction = .11). *P* for interaction were .42 and .15 for sex and age, respectively, of walking during commuting to work. Among participants with low work‐related physical activity, however, the OR of having depressive symptoms was lowest for ≥30 minutes of walking during commuting to work, although the reduction was not significant. The multivariable‐adjusted ORs (95% CIs) were 1.00 (reference), 0.94 (0.64, 1.39), 0.86 (0.48, 1.54), and 0.38 (0.14, 1.02) for none, <15 minutes, 15 to <30 minutes, ≥30 minutes of walking during commuting to work respectively (*P* for trend = .11), whereas the corresponding values were 1.00 (reference), 0.96 (0.63, 1.45), 0.68 (0.35, 1.33), and 1.71 (0.87, 3.34) among participants with high work‐related physical activity (*P* for trend = .59).

**TABLE 3:** Odds ratios of having depressive symptoms by walking during commuting to work (N = 2024)

	ORs (95% CI) by walking during commuting to work (min)				
	None	>0 to <15	15 to <30	≥30	*P* for trend^d^
Total population					
No. of subjects	1319	442	157	106	
No. of prevalent cases (%)	370 (28.1)	128 (29.0)	37 (23.6)	24 (22.6)	
Unadjusted model	1.00 (Reference)	1.05 (0.82, 1.33)	0.79 (0.54, 1.17)	0.75 (0.47, 1.20)	.19
Model 1^a^	1.00 (Reference)	0.87 (0.68, 1.12)	0.71 (0.48, 1.06)	0.73 (0.45, 1.17)	.039
Model 2^b^	1.00 (Reference)	0.96 (0.73, 1.27)	0.81 (0.53, 1.23)	0.85 (0.51, 1.43)	.32
Model 3^c^	1.00 (Reference)	0.96 (0.73, 1.27)	0.80 (0.52, 1.23)	0.95 (0.57, 1.60)	.48
Work‐related physical activity					
Low group					
No. of subjects	601	266	96	55	
No. of prevalent cases (%)	155 (25.8)	76 (28.6)	23 (24.0)	5 (9.1)	
Model 3^c^	1.00 (Reference)	0.94 (0.64, 1.39)	0.86 (0.48, 1.54)	0.38 (0.14, 1.02)	.11
High group					
No. of subjects	718	176	61	51	
No. of prevalent cases (%)	215 (29.9)	52 (29.5)	14 (23.0)	19 (37.3)	
Model 3^c^	1.00 (Reference)	0.96 (0.63, 1.45)	0.68 (0.35, 1.33)	1.71 (0.87, 3.34)	.59
	*P* for interaction^e^ = .11				

Abbreviations: BMI, body mass index; CI, confidence intervals; MET, Metabolic equivalent; OR, odds ratio.

^a^ Adjusted for age (in years, continuous), sex (male or female), site (factory A or B).

^b^ Adjusted for factors in Model 1 plus BMI (kg/m^2^, continuous), smoking (never smoker, quitter, current smoker consuming 1 to <20, or ≥20 cigarettes per day),alcohol consumption (non‐drinker, drinker consuming <1, 1 to <2, or ≥2 *go* of Japanese sake equivalent per day, where 1 *go* of Japanese sake contains approximately 23 g of ethanol), sleep duration on weekdays (<5 h per night, 5 to <6 h per night, 6 to <7 h per night, or ≥7 h per night), marital status (married or other), night or rotating shift work (yes or no), overtime work (<10 h per month, 10 to <30 h per month, or ≥30 h per month), job grade (low, middle, or high), job strain (quartile).

^c^ Adjusted for factors in Model 2 plus daily work‐related physical activity (MET‐h/d, continuous) and leisure‐time physical activity (none, >0 to <3.0, 3.0 to <10.0, ≥10.0 MET‐h/w).

^d^ The trend test were calculated for the associations between categories of walking during commuting to work converted into continuous variables and depressive symptoms.

^e^ Interaction between work‐related physical activity and walking during commuting to work was calculated by adding interaction terms in the model.

## 4 DISCUSSION

In the present study, leisure‐time physical activity was linearly and inversely associated with the prevalence of depressive symptoms, especially among workers with low work‐related physical activity. Longer walking time during commuting to work tended to be associated with lower prevalence of depressive symptoms among workers with low work‐related physical activity, however, this trend was not statistically significant. This is one of the few studies to show relations between walking to work and depressive symptoms, and the first study to show results stratified by work‐related physical activity among the Asian population.

A recent meta‐analysis reported that high leisure‐time physical activity was associated with a lower risk of depression.^8^ However, no study investigated whether leisure‐time physical activity‐depression relationship according to work‐related physical activity. We found that the association between leisure‐time physical activity and depressive symptoms was clearer among participants with low work‐related physical activity than among participants with high work‐related physical activity. This finding suggests that the impact of participating in leisure‐time physical activity on decreasing depressive symptoms might be stronger for sedentary workers. The levels of occupational physical activity of the participants may be a factor to be considered in planning the intervention at the workplace for mental well‐being.

We did not find a significant inverse association between the time spent in walking to work with having depressive symptoms. Existing studies have shown inconsistent results on this topic.^15^, ^16^ Interestingly, the present subgroup analysis showed that odds of depressive symptoms tended to decrease with increasing time spent walking to work among individuals who are less physically active, although the results were not statistically significant. Walking for 20 minutes is generally equivalent to walking a distance of about 1.5 km (this corresponds to a distance between subway stations in central Tokyo). Health experts sometimes recommend "walking from one station before your destination" for promoting physical health. Although convincing evidence is warranted, this recommendation might be also applied to mental health.

The strength of this study includes the availability of detailed information on physical activity and commuting activity, which allowed us to examine the associations stratified by work‐related physical activity. Our study has several limitations. First, the cross‐sectional design does not permit a distinction between cause and effect, and reverse causality may explain the present findings. Cohort and interventional studies are warranted to clarify the causality. To realize interventions, efforts in companies that are led by occupational health experts are needed in the future. Second, although we considered work‐related physical activity, we did not consider other potential confounders such as workplace environment (eg, variance in workload, interpersonal conflict), commuting means (eg, private car, train or bicycle). Furthermore, we did not include total commuting time (duration), due to the relatively high correlation with walking during commuting to work (Spearman's rank correlation = .34). However, we confirmed that the results did not change materially by including commuting time (data not shown). Third, physical activity levels and walking during commuting to work were assessed based on a self‐administered questionnaire. Future studies are needed using accelerometer‐based physical activity monitors for evaluation of physical activity or web‐based location information service for evaluation of the distance commuting to work. Fourth, although the intensity of physical activity may have an impact on depressive symptoms,^10^ we did not examine this issue for leisure‐time physical activity as the number of prevalent cases and participants were few in some categories when participants were divided by leisure‐time exercise intensity or volume and for commuting activity due to no information. Fifth, the depressive symptoms were assessed by self‐report. Clinically diagnosed depression should be considered in future studies. Sixth, since the study subjects participated voluntarily, selection bias may be a problem if non‐participation was associated with various physical activities or with depressive symptoms. Finally, since the participants were employees in a specific manufacturing company and its affiliates in Japan, caution should be exercised to generalize the present findings to workers with different backgrounds. The present results need to be confirmed in other occupations, industrial sectors, or regions.

In conclusion, the present cross‐sectional study supports evidence that leisure‐time physical activity is associated with less depressive symptoms among workers. Additionally, walking during commuting to work might be associated with decreasing depressive symptoms, especially in workers with relatively low physical activity or being sedentary on work, however, given the statistically non‐significant results, larger studies are needed to confirm the present findings.

## DISCLOSURE

*Approval of the research protocol*: The study protocol was approved by the ethics committee of the National Center for Global Health and Medicine, Japan. *Informed consent*: Written informed consent was obtained from each participant. *Registry and the registration no. of the study/trial*: N/A. *Animal studies*: N/A. *Conflict of interest*: The authors have no conflicts of interest directly relevant to the content of this article. TK, ME, IK are/were occupational physicians in the participating company.

## AUTHOR CONTRIBUTIONS

IK and TM conceived the ideas; KK, ME, TK, IK, and T. M. collected the data; KF, with support from KK and SC analyzed the data; and KF led the writing. All authors participated in critically reviewing the paper.

## Supporting information

Table S1
