# Supplementary material for: The association of leisure‐time physical activity and walking during commuting to work with depressive symptoms among Japanese workers: A cross‐sectional study
Source: J Occup Health. 2020 May 5;62(1):e12120. doi: 10.1002/1348-9585.12120 (PMC7199475; doi:10.1002/1348-9585.12120)
Supplement: Supplementary file 1 — Table S1 [file JOH2-62-e12120-s001.docx]

Supplementary Table 1. Characteristics of participant by walking during commuting to work (N = 2024)

|  | Walking during commuting to work (per one way) | | | |
| --- | --- | --- | --- | --- |
|  | None | >0 to < 15 min | 15 to < 30 min | ≥ 30 min |
| Number of participants | 1319 (65.2) | 442 (21.8) | 157 (7.8) | 106 (5.2) |
| Male sex | 1176 (89.2) | 385 (87.1) | 148 (94.3) | 100 (94.3) |
| Age, years | 43.1 ± 10.1 | 38.6 ± 10.0 | 42.5 ± 8.3 | 45.4 ± 9.1 |
| BMI, kg/m^2^ | 23.3 ± 3.4 | 22.9 ± 3.3 | 23.4 ± 3.4 | 22.9 ± 2.8 |
| CES-D score, points | 12.5 ± 7.6 | 12.4 ± 7.6 | 11.0 ± 7.3 | 10.5 ± 7.3 |
| Job strain, score | 0.49 ± 0.13 | 0.47 ± 0.11 | 0.46 ± 0.10 | 0.46 ± 0.10 |
| Current smoker | 428 (32.4) | 105 (23.8) | 35 (22.3) | 17 (16.0) |
| Heavy alcohol drinker^a^ | 114 (8.6) | 25 (5.7) | 16 (10.2) | 14 (13.2) |
| High job position | 346 (26.2) | 136 (30.8) | 85 (54.1) | 59 (55.7) |
| Shift work | 356 (27.0) | 36 (8.1) | 11 (7.0) | 6 (5.7) |
| Long overtime work (> 45 h/month) | 59 (4.5) | 21 (4.8) | 10 (6.4) | 6 (5.7) |
| Short sleep (< 5 h/day) | 94 (7.1) | 30 (6.8) | 17 (10.8) | 15 (14.2) |
| Married | 896 (67.9) | 241 (54.5) | 109 (69.4) | 90 (84.9) |
| High work-related physical activity^b^ | 718 (54.4) | 176 (39.8) | 61 (38.9) | 51 (48.1) |
| Leisure-time physical activity |  |  |  |  |
| None | 371 (28.1) | 108 (24.4) | 40 (25.5) | 13 (12.3) |
| > 0 to < 3.0 MET-h/w | 345 (26.2) | 123 (27.8) | 41 (26.1) | 18 (17.0) |
| 3.0 to < 10.0 MET-h/w | 289 (21.9) | 94 (21.3) | 31 (19.7) | 22 (20.8) |
| ≥ 10 MET-h/w | 314 (23.8) | 117 (26.5) | 45 (28.7) | 53 (50.0) |

Data are shown as mean ± standard deviation for continuous variables and number (percentages) for categorical variables.

BMI: body mass index, MET: metabolic equivalent, CES-D: Center for Epidemiologic Studies Depression.

^a^≥ 2 go of Japanese sake equivalent, 1 go of Japanese sake contains approximately 23 g of ethanol.

^b^≥ 7 MET-hours/day or more of work-related physical activity.
